# Targeting one‐carbon metabolism for cancer immunotherapy

**DOI:** 10.1002/ctm2.1521

**Published:** 2024-01-27

**Authors:** Xinxin Ren, Xiang Wang, Guowan Zheng, Shanshan Wang, Qiyue Wang, Mengnan Yuan, Tong Xu, Jiajie Xu, Ping Huang, Minghua Ge

**Affiliations:** ^1^ Department of Head and Neck Surgery Otolaryngology & Head and Neck Center, Cancer Center, Zhejiang Provincial People's Hospital (Affiliated People's Hospital) Hangzhou Medical College Hangzhou Zhejiang China; ^2^ Key Laboratory of Endocrine Gland Diseases of Zhejiang Province Hangzhou Zhejiang China; ^3^ Zhejiang Provincial Clinical Research Center for Malignant Tumor Hangzhou Zhejiang China; ^4^ Department of Pathology Cancer Center Zhejiang Provincial People's Hospital (Affiliated People's Hospital) Hangzhou Medical College Hangzhou Zhejiang China; ^5^ Department of Pharmacy Affiliated Hangzhou First People's Hospital Zhejiang University School of Medicine Hangzhou Zhejiang China; ^6^ Department of Pharmacy Center for Clinical Pharmacy Cancer Center, Zhejiang Provincial People's Hospital (Affiliated People's Hospital), Hangzhou Medical College Hangzhou Zhejiang China

**Keywords:** cancer immunotherapy, enzymes, inhibitors, one‐carbon metabolism

## Abstract

**Background:**

One‐carbon (1C) metabolism is a metabolic network that plays essential roles in biological reactions. In 1C metabolism, a series of nutrients are used to fuel metabolic pathways, including nucleotide metabolism, amino acid metabolism, cellular redox defence and epigenetic maintenance. At present, 1C metabolism is considered the hallmark of cancer. The 1C units obtained from the metabolic pathways increase the proliferation rate of cancer cells. In addition, anticancer drugs, such as methotrexate, which target 1C metabolism, have long been used in the clinic. In terms of immunotherapy, 1C metabolism has been used to explore biomarkers connected with immunotherapy response and immune‐related adverse events in patients.

**Methods:**

We collected numerous literatures to explain the roles of one‐carbon metabolism in cancer immunotherapy.

**Results:**

In this review, we focus on the important pathways in 1C metabolism and the function of 1C metabolism enzymes in cancer immunotherapy. Then, we summarise the inhibitors acting on 1C metabolism and their potential application on cancer immunotherapy. Finally, we provide a viewpoint and conclusion regarding the opportunities and challenges of targeting 1C metabolism for cancer immunotherapy in clinical practicability in the future.

**Conclusion:**

Targeting one‐carbon metabolism is useful for cancer immunotherapy.

## Graphical abstract

1. One carbon metabolism includes folate cycle, methionine cycle and transsulphuration pathway.

2. One carbon metabolism affects tumour microenvironment and the expression of immune checkpoint.

3. One carbon metabolism contributes to predict the prognosis of patients with immunotherapy and drug discovery.

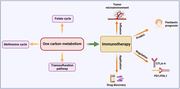

## 1 BACKGROUND

One‐carbon (1C) metabolism is a complex network of biological reactions that transfers 1C units to a variety of biosynthetic pathways.^1^ 1C metabolism could mediate the body's nutrient status by regulating the biosynthesis of proteins, lipids, DNA and other crucial substances for body homeostasis.^2^ In addition, 1C metabolism is essential for the biochemical process used to synthesise a series of metabolites, including amino acids, glutathione (GSH), nucleotides and S‐adenosylmethionine (SAM).^3^ Furthermore, 1C metabolism could participate in the energy balance by offering NADPH and ATP.^3^ Therefore, 1C metabolism not only transfers the 1C units between different biological processes but can also contribute to maintaining nutrient and redox conditions.

Accumulating evidence suggests that 1C metabolism is critical for cancer development based on the output it provides, as mentioned above. Certain amounts of nucleotides, proteins and lipids are needed for the rapid proliferation of cancer cells.^4^ The methylation of histones and DNA is the most frequent phenomenon when cancer occurs. Moreover, the redox state in the tumour microenvironment (TME) can easily influence the survival of cancer cells.^5^ In addition, 1C metabolism can be used as a target for cancer treatment. For example, methotrexate and 5‐fluorouracil (5‐FU), as 1C metabolic inhibitors, have been used for anticancer treatment in the clinic for over 50 years. Continuous anticancer drugs targeting 1C metabolism have emerged one by one, such as pemetrexed, pralatrexate and raltitrexed.^6^ These studies suggest that research on the relationship between 1C metabolism and cancer contributes to knowledge on the features of cancer development and new drug exploitation.

Cancer immunotherapy has become a hot topic in recent years due to its high efficiency, low treatment period and wide range of applications.^7^ Immunotherapy methods are extensively employed for various cancer treatments by using the patient's own immune system. At present, cancer immunotherapy plays a key role in combination therapy, including surgery, chemotherapy and radiotherapy.^8^ Immune checkpoints, the essential molecules expressed on immune cells, are used to inhibit the role of T lymphocytes and promote immune escape by suppressing the inhibitory signalling pathway and receptors in cancer.^9^ Over the years, immune checkpoint inhibitors, including pembrolizumab, atezolizumab and tremelimumab, which act on programmed cell death protein 1 (PD‐1), PD‐1 ligand 1 (PD‐L1) and cytotoxic T lymphocyte‐associated antigen 4 (CTLA‐4), respectively, have been widely used in the clinic.^10^ In addition, great progress has also been achieved in adoptive immunotherapy, chimeric antigen receptor (CAR) T‐cell therapy and tumour vaccines.^11^ Although the progression of immunotherapy has created a paradigm shift in cancer therapy, many patients do not benefit from immunotherapy. The significant distinction of patients’ response to immunotherapy and high frequency incidences of immune‐related adverse events (irAEs) obviously limited the clinical application of immunotherapy.^12^ On this basis, the expense of immunotherapy also hinders its clinical usage. Therefore, searching for effective targets for authenticating patients who benefit from immunotherapy is crucial for improving clinical satisfaction with immunotherapy.

1C metabolism plays essential roles in many cell types, including immune cells.^13^ Reports have shown that serine and glycine deprivation can decrease the proliferation of CD8^+^ T cells.^14^ In T cells, GSH is a crucial regulator of cellular redox homeostasis. Moreover, methionine deficiency could result in the destruction of cytokine secretion by CD4^+^ and CD8^+^ effector T cells.^15^, ^16^ In addition, cancer cells are capable of inhibiting antitumour immunity through competing for key nutrients and lowering the metabolic adaptability of immune cells.^17^ These phenomena demonstrate that 1C metabolism enables the mediation of antitumour immune responses by metabolic and nutrient sensing machinery.^18^ Therefore, methods that interfere with 1C metabolism provide a hopeful scheme for improving the application of immunotherapy.

In this review, we summarised the application of 1C metabolism in cancer immunotherapy, including the diverse influences of metabolic enzymes on immunotherapy and the potential clinical application of these enzyme inhibitors combined with immunotherapy. In addition, we examined the challenges and opportunities of targeting 1C metabolism in cancer.

## 2 COMPONENTS AND PATHWAYS OF 1C METABOLISM

**FIGURE 1:**
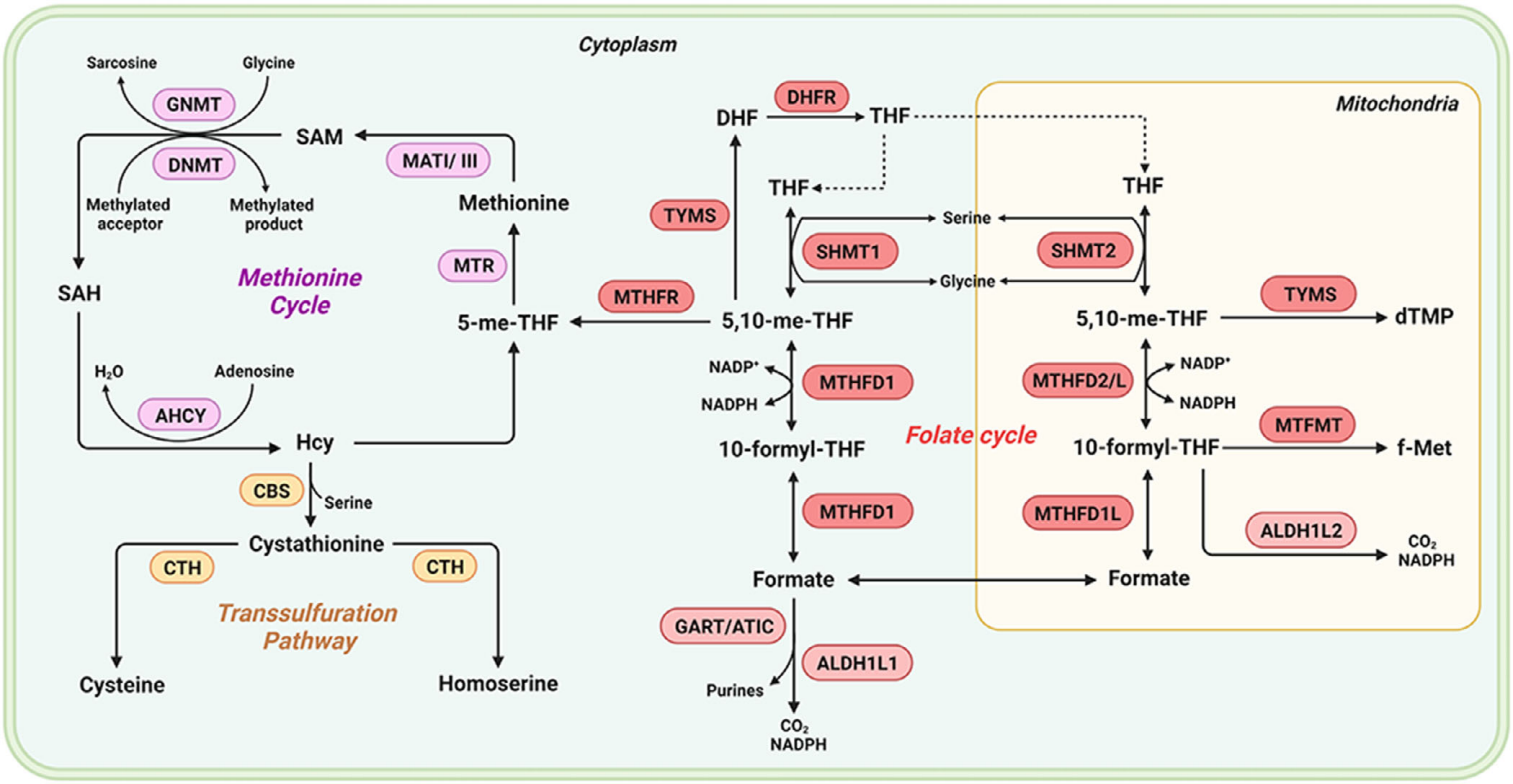
Overview of 1C metabolism metabolic pathways. Folate cycle metabolites are presented in red boxes. Methionine cycle metabolites are presented in purple boxes. Transsulphuration pathway metabolites are presented in yellow boxes. SHMT1, serine hydroxymethyl transferase 1; SHMT2, serine hydroxymethyl transferase 2; THF, tetrahydrofolate; DHF, dihydrofolate; TYMS, thymidylate synthetase; MTHFD1, methylenetetrahydrofolate dehydrogenase, cyclohydrolase and formyltetrahydrofolate synthetase 1; MTHFD1L, methylenetetrahydrofolate dehydrogenase 1 like; MTHFD2, methylenetetrahydrofolate dehydrogenase 2; MTHFD2L, methylenetetrahydrofolate dehydrogenase 2 like; ACHY, S‐adenosyl‐L‐homocysteine hydrolase; ALDH1L1, aldehyde dehydrogenase 1 family member L1; ALDH1L2, aldehyde dehydrogenase 1 family member L2; ATIC, 5‐aminoimidazole‐4‐carboxamide ribonucleotide formyltransferase/IMP cyclohydrolase; CBS, cystathionine β‐synthase; CTH, cystathionine γ‐lyase; DHF, dihydrofolate; DHFR, dihydrofolate reductase; DNMT, DNA methyltransferase; GART, phosphoribosylglycinamide formyltransferase; GNMT, glycine‐N‐methyltransferase; Hcy, homocysteine; MAT, methionine adenosyltransferase; MTFMT, mitochondrial methionyl‐tRNA formyltransferase; MTHFD1, methylenetetrahydrofolate dehydrogenase, cyclohydrolase and formyltetrahydrofolate synthetase 1; MTHFD1L, methylenetetrahydrofolate dehydrogenase 1 like; MTHFD2, methylenetetrahydrofolate dehydrogenase 2; MTHFD2L, methylenetetrahydrofolate dehydrogenase 2 like; MTHFR, methylenetetrahydrofolate reductase; MTR, methionine synthase; SAH, S‐adenosylhomocysteine; SAM, S‐adenosylmethionine; SHMT2, serine hydroxymethyl transferase 2; THF, tetrahydrofolate; TYMS, thymidylate synthetase.

1C metabolism mainly consists of the folate cycle, methionine cycle and transsulphuration pathway (Figure 1). 1C metabolism enzymes exist and function across three compartments, including the cytoplasm, mitochondria and nucleus. In the body, 1C metabolism promotes the transfer of 1C units in the form of methyl, methenyl, methylene and formyl groups to participate in the processes of cells. These processes include epigenetic control of genome function, essential molecule biosynthesis and energy homeostasis.

### 2.1 Folate cycle

The folate cycle plays a central role in 1C metabolism. Before it enters the folate cycle, dietary folate is reduced to dihydrofolate (DHF) by DHF reductase (DHFR).^19^ Then, DHF is further converted to tetrahydrofolate (THF), the biologically active form. In turn, THF is reversibly converted to 5,10‐methylenetetrahydrofolate by the B_6_‐dependent enzyme serine hydroxymethyltransferase (SHMT) and accompanied by the conversion of serine to glycine in both the cytoplasm and mitochondria.^20^ 5,10‐Methylenetetrahydrofolate can also be converted to THF through a succession of reactions mediated by methylenetetrahydrofolate dehydrogenases (MTHFD).^21^ Moreover, 5,10‐methylenetetrahydrofolate and THF can take part in the synthesis of purine through conversion to 10‐formyl‐THF.^22^ 5,10‐Methylenetetrahydrofolate can be reduced to 5‐methyltetrahydrofolate through B_2_‐dependent methylenetetrahydrofolate reductase (MTHFR), which supplies methyl groups for the methionine cycle.^3^

### 2.2 Methionine cycle

5‐Methyltetrahydrofolate transfers the methyl group to remethylate homocysteine (Hcy) for methionine formation, which is catalysed by the B12‐dependent enzyme methionine synthase (MTR).^23^ Then, methionine is adenylated by the enzyme methionine adenosyl‐transferase to SAM.^24^ SAM is the main methyl donor that helps maintain the normal function of cells by exerting its transmethylation function. In the process of methyl transfer, SAM is transformed to S‐adenosylhomocysteine (SAH) and then hydrolyzsd to Hcy and adenosine, which completes the methionine cycle.^25^

### 2.3 Transsulphuration pathway

The transsulphuration pathway contributes to the homeostasis of sulphur metabolism and redox mediation in cells.^26^ The pathway is used for cysteine production through the transfer of sulphur from Hcy to serine.^27^ Hcy and serine are catalysed by cystathionine β‐synthase to generate cystathionine.^28^ Then, cystathionine is converted to cysteine, which is catalysed by cystathionine γ‐lyase. The transsulphuration pathway is used for the biosynthesis of cysteine and protection of NADPH, which plays a central role in sustaining redox balance.^29^

### 2.4 1C metabolic pathways

Several pathways are involved in 1C metabolism. As a donor of the 1C unit, serine is essential for normal 1C metabolism. In the serine synthesis pathway (SSP), 3‐phosphoglycerate is converted into serine.^30^ Serine can also regulate the availability of 3‐phosphoglycerate in the SSP by activating PKM2.^31^ Studies have shown that serine supplementation contributes to cancer development by providing 1C units.^32^ Several SSP genes are upregulated in cancer cells. The use of inhibitors that target core SSP genes leads to reduced cancer growth.^31^ Therefore, serine‐derived 1C units may be essential for cancer progression.

Glycine is another source of 1C via the glycine cleavage system (GCS). This process catalyses the oxidation of glycine and cleaves a methylene group from glycine to form methylene‐THF.^33^ Studies have indicated that glycine and the GCS contribute to tumourigenesis. The reduction of glycine levels and suppression of the GCS can inhibit the proliferation and growth of cancer cells.^34^ Furthermore, choline is also a source of 1C through its conversion to betaine. Betaine is used to synthesise methionine from Hcy and is transformed into dimethylglycine.^35^ Dimethylglycine provides a 1C unit to THF to form methenyl‐THF. Additionally, 1C units are obtained from histidine and tryptophan, which are used for the synthesis of methenyl‐THF and formyl‐THF, respectively.^36^

## 3 APPLICATION OF 1C METABOLISM IN CANCER IMMUNOTHERAPY

**FIGURE 2:**
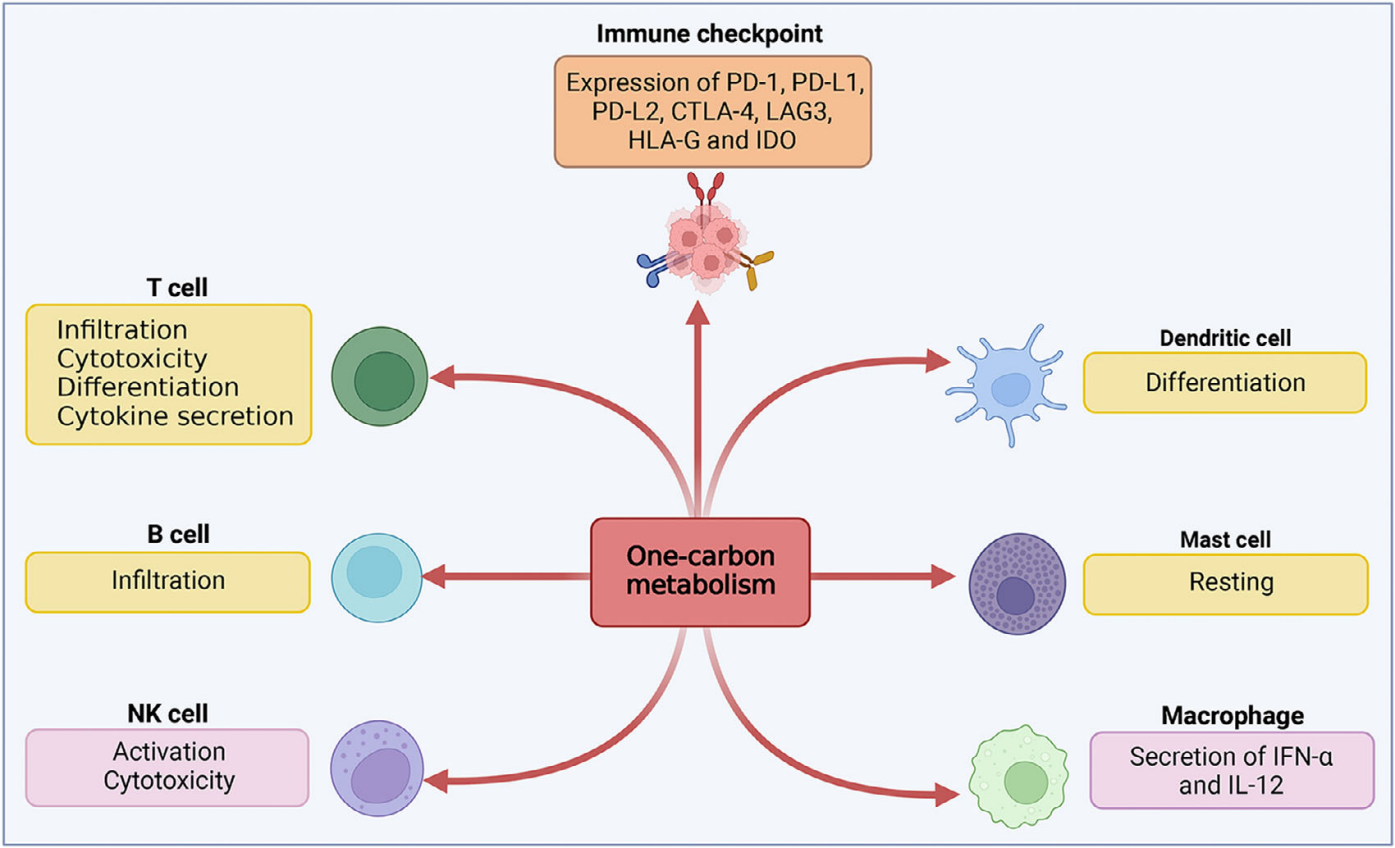
The potential function of 1C metabolism enzymes on cancer immunotherapy. 1C metabolism enzymes could affect cancer immunotherapy by influencing the state of immune cells and the expression of immune checkpoints. Immune cells including T cell, B cell, natural killer (NK) cell, dendritic cell, mast cell and macrophage. Immune checkpoints including PD‐1, PD‐L1, PD‐L2, CTLA‐4, LAG3, HLA‐G and IDO. CTLA‐4, cytotoxic T lymphocyte‐associated antigen 4; HLA‐G, human leukocyte antigen G; IDO, indoleamine 2,3‐dioxygenase; IFN‐α, interferon‐α.IL, interleukin; LAG3, lymphocyte activating 3.

1C metabolism maintains biosynthesis and metabolic regulation to preserve the function of immune cells. Reports have shown that 1C metabolism plays an essential role in cancer immunotherapy, such as the prediction of patient response. Thus, in this section, we discuss the roles of 1C metabolic enzymes in cancer immunotherapy (Figure 2 and Table 1).

**TABLE 1:** Application of 1C metabolism enzymes in cancer immunotherapy.

Enzyme	Target	State	Correlation	Cancer	References
SHMT2	CD4+ and CD56 T cell, immature B cell	Infiltration	Negative	LUAD	^37^
	Treg, myeloid‐derived suppressor cells	Infiltration	Positive	RCC	^38^
MTHFD1L	PD‐1	Expression	Negative	Colorectal cancer	^39^
MTHFD2	Mast and NK cell	Activation/resting	Positive	HNSCC	^40^
	CTLA4, TIGIT, CD276, HAVCR2, PDCD1, LAG3	Expression	Positive/negative	UBC	^41^
	PD‐L1	Expression	Positive	Pancreatic cancer	^42^
	Treg and Th17 cell	Infiltration	Positive	OSCC	^43^
	Treg cell	Differentiation	Negative	NA	^44^
TYMS	MHC class I peptide	Presentation	Negative	DLBCL	^45^
	Peripheral T cell	Division	Positive	DLBCL	^45^
	PD‐L1, PD‐L2, CTLA‐4, PD‐1	Expression	Positive	NSCLC	^46^
DNMT1	CXCL9, CXCL10, CXCL12	Expression	Negative	Ovarian cancer	^47^, ^48^
	PD‐1, PD‐L1	Expression	Positive	HNSCC, ovarian cancer	^49^
	T cell	Infiltration	Positive	HNSCC, ovarian cancer	^49^
	HLA‐G, IDO	Expression	Negative	Solid tumours CSCC	^50^, ^51^
GNMT	Treg cell	Differentiation	Negative	HCC	^52^

Abbreviations: CSCC, cutaneous squamous cell cancer; DLBCL, diffuse large B‐cell lymphomas; HCC, hepatocellular carcinoma; HLA‐G, human leukocyte antigen G; HNSCC, head and neck squamous cell carcinoma; IDO, indoleamine 2,3‐dioxygenase; LUAD, lung adenocarcinoma; NSCLC, non‐small cell lung cancer; OSCC, oral squamous cell carcinoma; RCC, renal cell carcinoma; UBC, urothelial bladder carcinomas.

### 3.1 Serine hydroxymethyltransferase

SHMT is the primary mediator of 1C metabolism, which converts serine and THF to glycine and 5,10‐CH_2_‐THF, respectively.^53^ SHMT includes two isoforms, SHMT1 and SHMT2, which perform functions in the cytoplasm and mitochondria, respectively. Serine is a crucial metabolite for the proliferation and response of T cells.^54^ Thus, SHMT1 and SHMT2 could be regarded as biosynthetic checkpoints for T‐cell growth. A common single‐nucleotide polymorphism, cytosine (C) to thymus pyrimidine (T), occurs at nucleotide 1420 of the SHMT1 gene (C1420T, rs1979277).^55^ This change could result in leucine‐to‐phenylalanine amino acid substitution in SHMT1.^55^ The SHMT1 C1420T polymorphism can decrease enzyme activity and lead to a reduction in folate levels, Hcy re‐methylation and DNA synthesis.^56^ Studies have shown that SHMT1 1420CT/TT could decrease the risk of acute lymphocytic leukaemia.^57^, ^58^ This suggests that deletion and reduction of SHMT1 activity may affect the function of the immune system. In addition, the expression of SHMT2 was negatively related to tumour‐infiltrating lymphocytes, including CD4^+^ T cells, CD56 natural killer cells, Th17 cells and immature B cells, in lung adenocarcinoma (LUAD).^37^ Moreover, SHMT2 levels were positively associated with the infiltration of myeloid‐derived suppressor cells and Treg cells, which resulted in accelerated immune escape in papillary renal cell carcinoma.^38^ Therefore, SHMT was connected to cancer immunotherapy by influencing the TME. SHMT1 and SHMT2 are potential biomarkers for immunotherapy.

### 3.2 Methylenetetrahydrofolate dehydrogenases

MTHFD family genes are vital enzymes in 1C metabolism that are involved in oxidative stress and nucleic acid synthesis.^59^ The MTHFD family includes four isoforms, MTHFD1, MTHFD1L, MTHFD2 and MTHFD2L.^60^ In the mitochondria, MTHFD2 and MTHFD2L convert 5,10‐CH_2_‐THF to 10‐f‐THF. Then, 10‐f‐THF is converted to formate via MTHFD1L catalysis. In the cytoplasm, formate is catalysed by MTHFD1 to form 10‐f‐THF and 5,10‐CH_2_‐THF. Recent reports have revealed that MTHFD family genes are associated with immunotherapy. Mutations in MTHFD1 could result in the occurrence of severe combined immunodeficiency.^61^ The expression of MTHFD1L was upregulated in colon cancer cells treated with anti‐PD‐1 nivolumab, which promoted the proliferation of cancer cells.^39^ In addition, MTHFD2 upregulation was positively related to mast cell activation and NK cell resting in head and neck squamous cell carcinoma, which is an immune‐related metabolic enzyme.^40^ Moreover, MTHFD2 is significantly related to the prognosis of patient and the expression of immune checkpoints. Unsupervised clustering based on many genes, including MTHFD2, contributed to predicting the response of gastric cancer patients to immunotherapy.^62^ MTHFD2 expression was significantly connected to many immune checkpoints, including CTLA4, TIGIT, PD‐L1, CD276, HAVCR2, PDCD1 and LAG3, in urothelial carcinomas of the bladder.^41^ MTHFD2 promoted the expression of PD‐L1, which led to the immune evasion of pancreatic cancer.^42^ Furthermore, MTHFD family genes were discovered to be obviously positively correlated with tumour‐infiltrating immune cells, including Treg and Th17 cells, in oral squamous cell carcinoma.^43^ Given that MTHFD2 regulated de novo purine synthesis, it was proved to promote the proliferation, differentiation and inflammatory cytokine production of T cells, which had been regarded as a metabolic checkpoint controlling the fate and function of T cells.^44^ These studies illustrated that MTHFD family genes show great promise for applications in immunotherapy.

### 3.3 Thymidylate synthase

Thymidylate synthase (TYMS) was used for DNA synthesis and repair. In the process of 1C metabolism, TYMS converts deoxyuridylate to deoxythymidine‐5′‐monophosphate in a 5,10‐methylene‐THF‐dependent manner.^63^ Many drugs have been developed by targeting TYMS, including 5‐FU, pemetrexed and raltitrexed, which have already been applied in the clinic for years. Additionally, TYMS should be a potential target for immunotherapy. First, TYMS was negatively associated with MHC class I peptide presentation in diffuse large B‐cell lymphomas. TYMS inhibition contributed to the deletion of dividing peripheral T cells.^45^ In addition, TYMS expression is associated with immune checkpoint level and therapeutic response. TYMS positivity was related to PD‐L1 expression across most cancer types.^46^ The prognostic signature based on metabolism‐related genes, including TYMS, contributed to predicting the expression of immune checkpoints and patient response to immunotherapy in LUAD and prostate cancer.^64^, ^65^ Cytokine‐induced killer cells, which are commonly used for adoptive immunotherapy, suppress the expression of TYMS and improve the drug sensitivity of breast cancer cells.^66^ Pemetrexed downregulated the level of PD‐L1 by inactivating TYMS and provided a favourable microenvironment for immunotherapy of NSCLC.^67^ In a randomised clinical trial, TYMS upregulation was correlated with poorer prognosis of colon cancer patients receiving immunotherapy.^68^

### 3.4 DNA methyltransferase 1

DNA methyltransferase 1 (DNMT1) plays a central role in maintaining patterns of DNA methylation. In 1C metabolism, DNMT1 catalyses the transfer of the methyl group of SAM to the CpG dinucleotides of the gene promoter, and SAM is changed to SAH.^69^ DNMT1 deficiency blocks immune cell infiltration and the innate immune response.^70^ Peng et al. demonstrated that DNMT1 inhibited the expression of CXCL9, CXCL10 and CXCL12 and in turn transferred effector T cells to the TME in ovarian cancer.^47^, ^48^ A study showed that DNMT1 might block the epigenetics of immune protective genes and then influence the clinical response to immunotherapy. Moreover, the level of DNMT1 is connected to the state of immune cell and immune checkpoint expression. The expression of DNMT1 was positively related to PD‐1/PD‐L1 levels and T‐cell infiltration.^49^ Suppression of DNMT1 could result in the upregulation of the immune checkpoint proteins human leukocyte antigen G and indoleamine 2,3‐dioxygenase, which provided targets for cancer immunotherapy.^50^, ^51^ Treatment with DNMT1 inhibitors, such as DZNep, could enhance effector T‐cell tumour infiltration and increase the response of patients to PD‐1/PD‐L1 inhibitors and adoptive T‐cell transfusion.^47^, ^71^, ^72^, ^73^, ^74^, ^75^ In conclusion, DNMT1 might be a promising target for cancer immunotherapy.

### 3.5 Methylenetetrahydrofolate reductase

MTHFR is the rate‐limiting enzyme that catalyses the conversion of 5,10‐methylenetetrahydrofolate to 5‐methyltetrahydrofolate.^76^, ^77^ A study showed that increasing the concentration of unmetabolised folic acid could decrease the number and cytotoxicity of NK cells.^78^ In addition, the polymorphisms of MTHFR could influence the regulation of immune cells. The single nucleotide polymorphisms C677T and A1298C are two common variants of MTHFR that significantly decrease the activity of MTHFR.^79^ The MTHFR C677T gene polymorphism is derived from the replacement of cytosine (C) with thymus pyrimidine (T) at position 677, resulting in an alanine‐to‐valine substitution.^80^ MTHFR 677CT and MTHFR 677TT showed 70% and 35% MTHFR activity, respectively, compared to that of the control.^81^ The MTHFR A1298C gene polymorphism is a substitution of adenine (A) with cytosine (C) at nucleotide 1298, resulting in the conversion of glutamate to alanine.^82^ This phenomenon reduced the enzymatic activity of MTHFR by 65−70%.^83^ These two mutations decrease the enzymatic activity of MTHFR and lead to folate synthesis disorders, elevated Hcy levels and abnormal DNA synthesis and methylation.^84^ MTHFR polymorphism influenced the level of interleukin‐6 by affecting plasma Hcy levels, which are involved in innate immune regulation.^85^ These results suggested that MTHFR polymorphisms might be connected to the body immune state. Moreover, reports also revealed that MTHFR C677T or A1298C could affect the immune response and was associated with the risk of breast cancer and acute lymphoblastic leukaemia.^86^ In addition, MTHFR contributed to predicting the immunotherapy response. In a retrospective study, MTHFR A1298C might affect the overall response rate of non‐Hodgkin lymphoma patients during rituximab treatment.^87^

### 3.6 DHF reductase

For the coenzyme factor NADPH, DHFR catalyses the reduction of DHF to THF. The method of targeting DHFR for cancer treatment has been used for decades. Methotrexate showed anticancer function and immune inhibition through focusing on DHFR. At present, DHFR has been reported to participate in immunotherapy. During CAR T‐cell therapy, DHFR was fused to CAR and then targeted CD19, which improved the efficacy of CAR T cells.^88^ In addition, Birocchi et al.^89^ constructed an IFN‐α‐DHFR delivery system based on the fusion of the DHFR mutant and IFN‐α, which was beneficial for improving the microenvironment of glioblastoma.

### 3.7 Aldehyde dehydrogenase 1 family member L1

Aldehyde dehydrogenase 1 family member L1 (ALDH1L1) catalyses the decomposition of 10‐f‐THF into carbon dioxide and NADPH, which are present in the cytoplasm.^90^ A study showed that the expression of ALDH1L1 was associated with infiltrating immune cells.^91^ Through a cluster model based on ALDH1L1, prediction can be made regarding immune infiltration and drug sensitivities to immune checkpoint inhibitors in clear cell renal cell carcinoma.^92^

### 3.8 Glycine N‐methyltransferase

The main function of glycine N‐methyltransferase (GNMT) is to catalyse SAM conversion to SAH while promoting glycine to form sarcosine, which regulates the availability of methyl groups in cells.^93^ As a tumour suppressor gene, GNMT is essential for the maintenance of immune functions. Eudy et al.^94^ found that GNMT deletion disrupted adequate immune cell activation. In addition, GNMT deficiency strengthened Treg cell differentiation and regulated the function of CD4^+^ T cells.^52^

### 3.9 SAH hydrolase

SAH hydrolase (ACHY) plays a crucial role in cellular methylation maintenance by catalysing the hydrolysis of SAH to Hcy and adenosine. SAH suppressed processes that modify methylation through negative feedback, which affected lymphocyte function and the immune response. Additionally, ACHY might participate in dendritic cell differentiation.^95^ Thus, abnormal ACHY was associated with immunotherapy. Unsupervised clustering based on NF‐κB‐related metabolic genes, including ACHY, could be applied to predict the prognosis and response to immunotherapy of gastric cancer patients.^62^

## 4 TARGET 1C METABOLISM ENZYMES FOR CANCER IMMUNOTHERAPY

**FIGURE 3:**
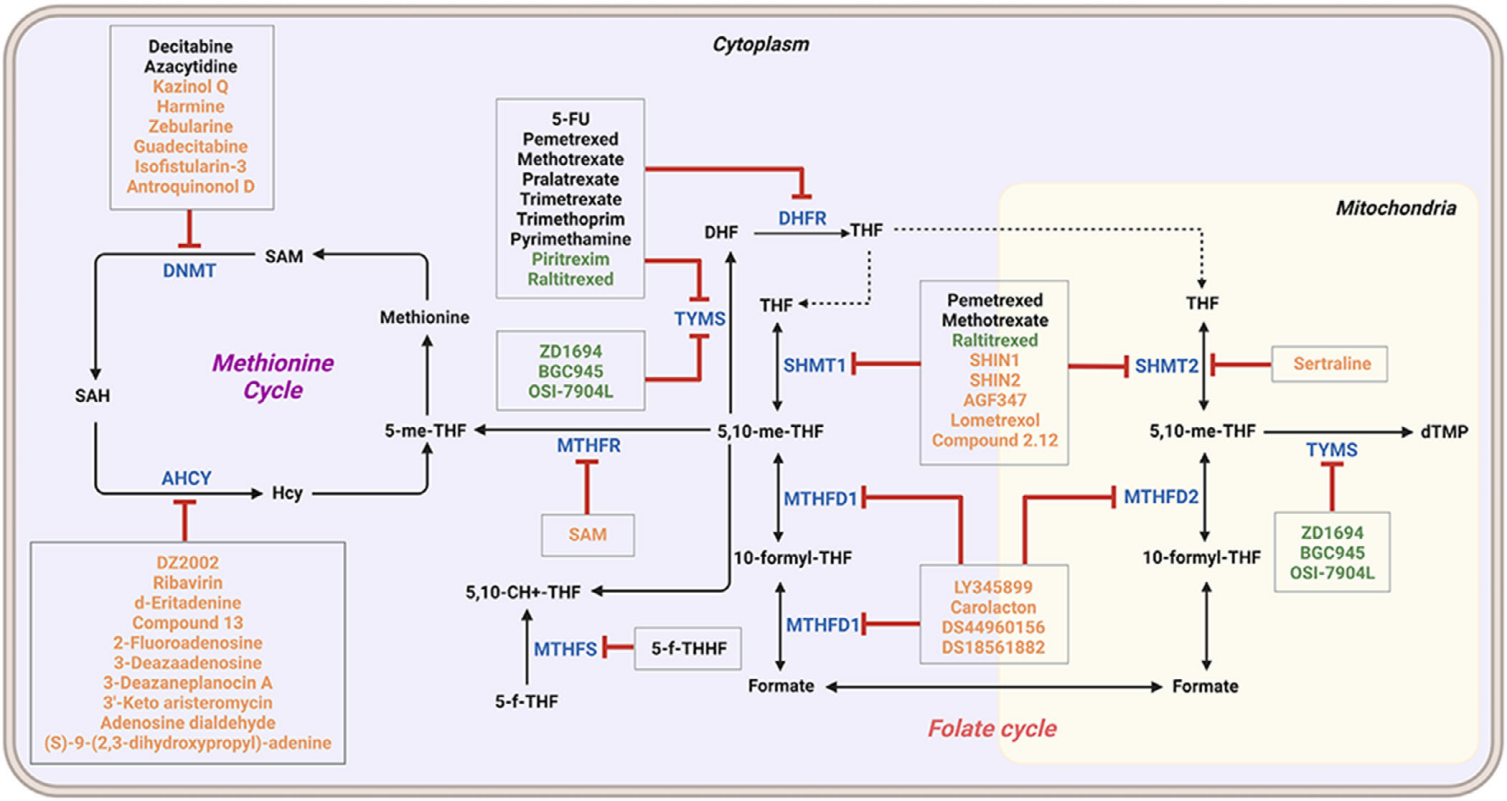
Summary of the inhibitors targeting 1C metabolism. Colouring: US FDA‐approved trials are shown in black; clinical trials are shown in green; preclinical trials are shown in yellow.

**FIGURE 4:**
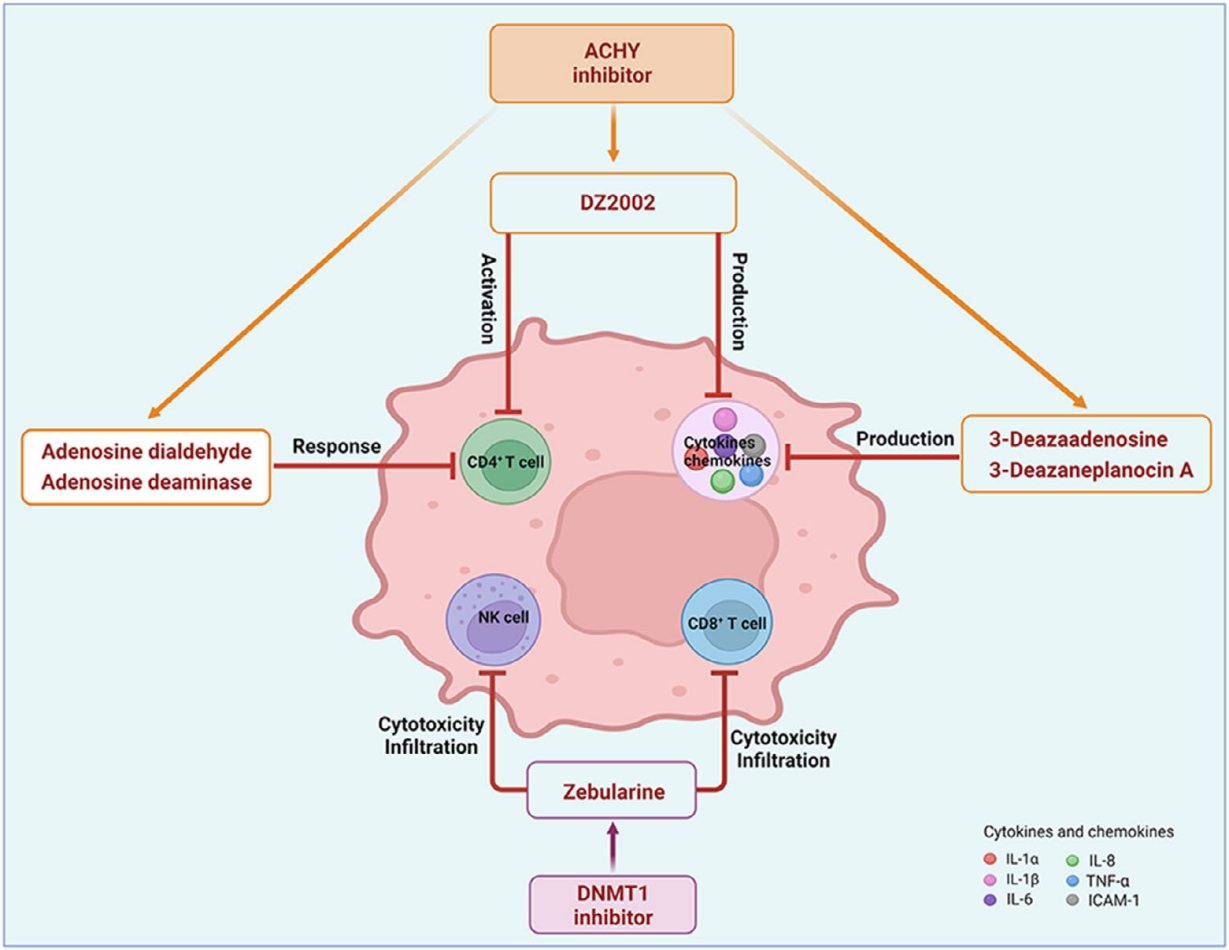
The application of 1C metabolism inhibitors on cancer immunotherapy. 1C metabolism inhibitors could affect cancer immunotherapy by influencing the state of immune cells and the production of cytokines and chemokines. Immune cells including CD4^+^ T cell, CD8^+^ T cell and NK cell. Cytokines and chemokines including IL‐1α, IL‐1β, IL‐6, IL‐8, TNF‐α and ICAM‐1. ICAM‐1, intercellular adhesion molecule‐1: TNF‐α, tumour necrosis factor‐α

1C metabolism is closely related to cancer immunotherapy, as mentioned above. Targeting metabolic enzymes is a promising method for improving cancer immunotherapy. Then, we summarised the inhibitors of these metabolic enzymes and the potential application of inhibitors in cancer immunotherapy (Figures 3 and 4).

### 4.1 SHMT inhibitors

SHMT1 and SHMT2 are the key enzymes in 1C metabolism and have been studied as cancer therapeutic targets. In addition, serine is a crucial metabolite for effector T cell expansion, which influence the immune condition of the body.^54^ Thus, the development of SHMT1/2 inhibitors contributed to enriching clinical medication. Triazine antifolate NSC127755 was the first inhibitor of SHMT.^96^ However, the development of the inhibitor was limited due to the adverse effects and structural instability in vivo. Recent studies have focused on pyrazolopyrans, which presented excellent inhibitory effects on SHMT. Compound 2.12, SHIN1 and SHIN2, as plant‐derived compounds, were authenticated as inhibitors of SHMT1/2.^97^, ^98^ The folate analogue of AGF347 is known to exhibit a dual inhibitory function on SHMT1/2.^99^ In addition, pemetrexed, lometrexol and sertraline, which have been used in the clinic, could also suppress the activity of SHMT2.^100^, ^101^, ^102^, ^103^, ^104^

Moreover, these inhibitors also showed powerful anticancer effects. At the time of initial discovery, SHIN1 was found to suppress the formation of colon cancer tumour xenografts. SHIN2 inhibited the proliferation and growth of T‐cell acute lymphoblastic leukaemia (T‐ALL) and Burkitt lymphoma cells and synergised with methotrexate to exert anticancer effects.^105^, ^106^ SHIN1 and SHIN2 could improve the survival of T‐ALL and Burkitt lymphoma cells in vivo.^105^, ^106^, ^107^ Compound 2.12 promoted the death of lung cancer cells by inducing apoptosis.^108^ Dekhne et al discovered that AGF347 inhibited the growth of pancreatic tumour xenografts and showed in vivo anticancer activity. Additionally, the antidepressant sertraline presented a new function in the inhibition of breast cancer growth by inducing G1‐S cell cycle arrest^102^ (Figure 3 and Table 2).

**TABLE 2:** Inhibitors of human SHMTs.

Inhibitor	Target	Structure	Cancer	Cell lines	Activity	References
Compound 2.12	SHMT1	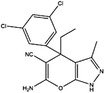	LUAD	H1299	IC_50_ = 0.65 μM	^97^, ^98^
	SHMT2				IC_50_ = 1.4 μM	
SHIN1	SHMT1	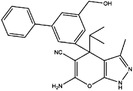	Colorectal cancer	HCT116	IC_50_ = 5 nM	^98^
	SHMT2				IC_50_ = 13 nM	
SHIN2	SHMT1	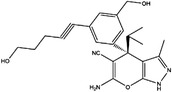	ALL	MOLT‐4	NA	^98^
	SHMT2					
AGF347	SHMT1	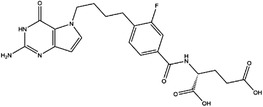	Pancreatic cancer	PaCa‐2	Ki = 2.91 μM	^99^, ^108^
	SHMT2				Ki = 2.19 μM	
Pemetrexed	SHMT1	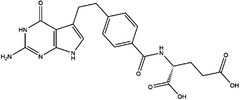	Malignant pleural mesothelioma and NSCLC	NA	Ki = 71.9 ± 11.6 μM	^103^
	SHMT2				Ki = 79.2 ± 6.8 μM	
Lometrexol	SHMT1	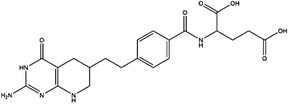	Leukaemia, breast cancer	CCRF‐CEM, BT549, MDA‐MB‐231	Ki = 36.5 ± 4.1 μM	^103^
	SHMT2				Ki = 49.7 ± 4.7 μM	
Methotrexate	SHMT1	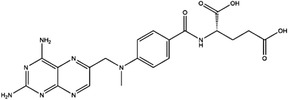	ALL and solid tumours	NA	Ki = 59.1 ± 8.2 μM	^103^
	SHMT2				Ki = 87.4 ± 8.3 μM	
Raltitrexed	SHMT1	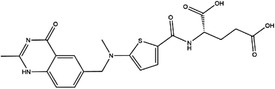	Colorectal cancer	NA	Ki = 72.7 ± 5.9 μM	^103^
	SHMT2				Ki = 94.0 ± 11.5 μM	
Sertraline	SHMT2	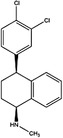	Lung, colorectal, breast, liver, ovarian and oral cancer	A549, H522, PC9/R and H1975, HCT116, HT‐29, MCF‐7, HepG2, OVCAR8, OC2	Ki = 13.1 μM	^102^, ^104^

Abbreviations: ALL, acute lymphocytic leukaemia; LUAD, lung adenocarcinoma.

### 4.2 MTHFD inhibitors

Increasing evidence has revealed the clinical significance of MTHFD2, which has attracted attention in the development of MTHFD2‐targeted drugs.^109^ At present, four compounds that act on MTHFD1/2 have been reported. The first inhibitor, LY345899, is a folate analogue that lacks selectivity for MTHFD1/2.^110^ At approximately the same time, the natural product carolacton, which is an inhibitor of FolD, presented its novel function by suppressing MTHFD1/2.^111^ Surprisingly, carolacton was selective for MTHFD1/2, which influenced the cyclohydrolase and dehydrogenase functions, respectively.^111^, ^112^ Recently, DS44960156 and DS18561882 were developed with a better selectivity for MTHFD2 and preferable in vivo function.^113^, ^114^

Regarding anticancer function, LY345899 inhibited the growth and metastasis of colorectal cancer.^115^ Carolacton can suppress the growth of various cancer cells, such as human epidermoid carcinoma KB‐3.1 cells.^111^, ^116^, ^117^, ^118^ In addition, oral administration of DS18561882 could obviously suppress the proliferation of breast cancer, which has potential clinical application value.^114^ In castration‐resistant prostate cancer, DS18561882 cooperated with enzalutamide to inhibit the proliferation of cancer cells in vitro and in vivo^119^ (Figure 3 and Table 3).

**TABLE 3:** Inhibitors of human MTHFDs.

Inhibitor	Target	Structure	Cancer	Cell lines	IC_50_	References
LY345899	MTHFD1	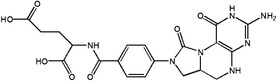	Colorectal cancer	SW620	96 nM	^110^
	MTHFD2				0.663 μM	
Carolacton	MTHFD1	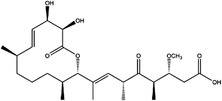	Colorectal, liver, lung and breast cancer	HCT116, Huh7, A549, MCF‐7	38 nM	^111^, ^112^ ^]^
	MTHFD2				6.5 nM	
DS44960156	MTHFD1	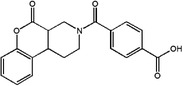	NA	NA	>30 μM	^113^
	MTHFD2				1.6 μM	
DS18561882	MTHFD1	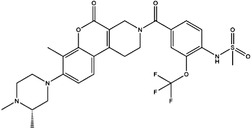	Breast cancer	MDA‐MB‐231	0.57 μM	^114^
	MTHFD2				6.3 nM	

### 4.3 TYMS and DHFR inhibitors

TYMS and DHFR are the earliest 1C metabolism enzymes and clinical targets that have been successfully applied in cancer treatment. At present, except for 5‐FU, the inhibitors of TYMS and DHFR are folate derivatives, including methotrexate, raltitrexed, pemetrexed, pralatrexate, trimetrexate, trimethoprim and pyrimethamine.^120^, ^121^, ^122^, ^123^, ^124^, ^125^, ^126^, ^127^, ^128^, ^129^ In addition to inhibitors that have been already used in the clinic, there are also some compounds in clinical trials, including piritrexim, BGC945, ZD1694 and OSI‐7904L.^130^ For anticancer function, piritrexim has been studied in clinical trials alone and combined with other drugs in various cancers.^131^, ^132^, ^133^, ^134^ BGC945 causes a strong effect on folate receptor‐overexpressing human cancer cells.^135^ In a phase II clinical trial, OSI‐7904L administration was selected for patients with refractory solid tumours^136^ (Figure 3 and Table 4).

**TABLE 4:** 1C metabolism enzyme inhibitors.

Enzyme	Inhibitor	Structure	Status	Cancer	Cell line	Activity	References
TYMS	5‐FU	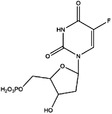	US FDA‐approved drug	Breast, pancreatic, gastric and colorectal cancer	NA	Ki = 0.2 nM	^123^
	Raltitrexed	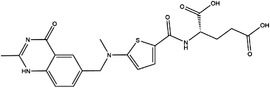	EMA‐approved drug	Colorectal cancer	NA	Ki = 62 nM	^124^
	Pemetrexed	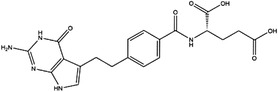	US FDA‐approved drug	Malignant pleural mesothelioma and NSCLC	NA	Ki = 109 nM	^125^
	BGC945	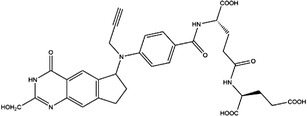	Preclinical	CSCC, oral epidermoid carcinoma, ovarian cancer and human choriocarcinoma	A431, A431‐FBP, KB, IGROV‐1 and JEG‐3	Ki = 1.2 nM	^135^
	ZD1694	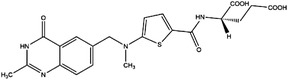	Preclinical	Ovarian, breast and colorectal cancer, leukaemia, head and neck cancer	CH1, 41 M, MCF‐7, HT29, MOLT‐3, K562, A253, FaDu	Ki = 60 nM	^130^
	OSI‐7904L	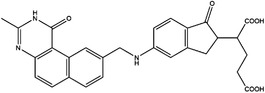	Phase I and II	Advanced solid tumours colorectal, gastric and advanced biliary cancer	NA	Ki = 0.09 nM	^136^
DHFR	Methotrexate	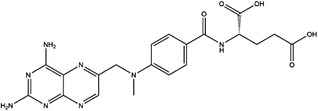	US FDA‐approved drug	ALL and solid tumours	NA	Ki = 0.0055 nM	^126^
	Raltitrexed	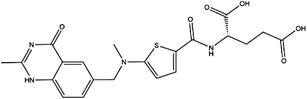	EMA‐approved drug	See above for TYMS	NA	Ki = 92 nM	^124^
	Pemetrexed	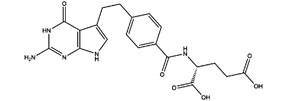	US FDA‐approved drug	See above for TYMS	NA	Ki = 7 nM	^125^
	Pralatrexate	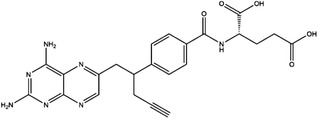	US FDA‐approved drug	Peripheral T cell lymphomas	NA	Ki = 0.0134 nM	^128^
	Trimetrexate	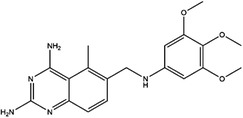	US FDA‐approved drug	NSCLC and pleural mesothelioma	NA	IC_50_ = 80.9 nM	^121^
	Trimethoprim	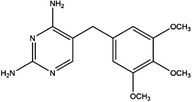	US FDA‐approved drug	NA	NA	IC_50_ = 20 nM	^129^
	Pyrimethamine	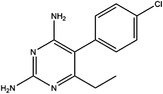	US FDA‐approved drug	Colorectal, ovarian, breast and prostate cancer	HCT116, SW480, A2780, SKOV3, TUBO, TM40B‐MD, DU145 and PC3	IC_50_ = 0.53 μM	^122^
	Piritrexim	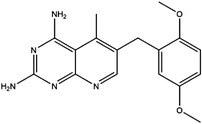	Phase II	Bladder cancer	NA	IC_50_ = 5 nM	^134^
AHCY	DZ2002	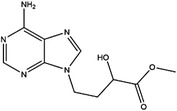	NA	NA	NA	Ki = 17.9 nM	^137^
	3‐Deazaadenosine	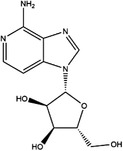	Preclinical	Breast and liver cancer, leukaemia	MCF7, MDA‐MB‐231, SKBr3, HepG2, HL‐60, U‐937	IC_50_ = 20 μM	^133^, ^134^
	3‐Deazaneplanocin A	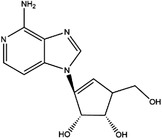	Preclinical	Colorectal cancer	HCT116, HT‐29	Ki = 23 nM	^138^, ^139^
	d‐Eritadenine	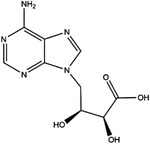	NA	NA	NA	IC_50_ = 30 nM	^140^
	(S)−9‐(2,3‐dihydroxypropyl)‐adenine	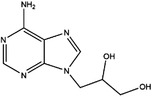	NA	NA	NA	IC_50_ = 24 μM	^140^
	Ribavirin	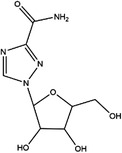	Preclinical	Lung, ovarian, liver, thyroid cancer	NCI‐H69, H3255, A549, OVCAR3, Huh‐7, HepG2 8505C, FTC‐133	IC_50_ = 0−5 mM	^141^, ^142^, ^143^, ^144^, ^145^, ^146^
	3′‐Keto aristeromycin	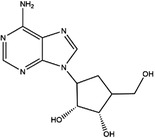	NA	NA	NA	IC_50_ = 0.2 μM	^147^
	2‐Fluoroadenosine	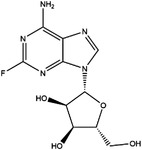	NA	NA	NA	IC_50_ = 66 μM	^147^
	Adenosine dialdehyde	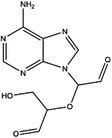	Preclinical	Prostate, oral, breast, glioma cancer	LNCaP, 22Rv1, SAS, OECM‐1, HSC‐3 MDA‐MB‐231, MCF‐7, U87	IC_50_ = 40 nM	^147^, ^148^, ^149^, ^150^, ^151^
	Compound 13	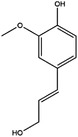	Preclinical	Breast and prostate cancer	MDA‐MB 231, MCF‐7, LNCaP	IC_50_ = 34 nM	^152^, ^153^, ^154^
DNMT1	Azacytidine	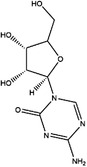	US FDA‐approved drug	Breast and colorectal cancer, leukaemia	NA	IC_50_ < 20 μM	^155^
	Decitabine	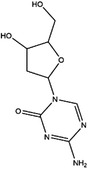	US FDA‐approved drug	Leukaemia	NA	IC_50_ = 2.6 μM	^156^
	Guadecitabine	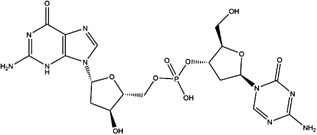	Phase I and II	Ovarian and breast cancer	NA	IC_50_ < 5 μM	^157^
	Antroquinonol D	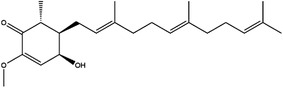	Preclinical	Breast and pancreatic cancer, NSCLC, glioma	MDA‐MB‐231, PANC‐1, AsPC‐1, A549, C6	IC_50_ < 5 μM	^69^, ^158^, ^159^, ^160^
	Isofistularin‐3	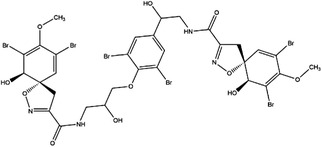	Preclinical	Pheochromocytoma, lymphoma, leukaemia, prostate cancer	PC12, RAJI, U‐937, JURKAT, K‐562, HL‐60, PC‐3	IC_50_ = 8.1–18.9 μM	^69^, ^161^, ^162^
	Kazinol Q	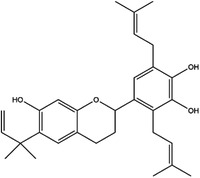	Preclinical	Prostate and breast cancer	LNCaP, MCF‐7	IC_50_ = 7 μM	^69^, ^163^
	Harmine	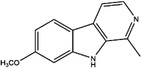	Preclinical	Breast, cervical, liver and pancreatic cancer	MDA‐MB‐231, MCF‐7, HeLa, HepG2, Hep3B, BxPC‐3, CFPAC‐1, SW1990, PANC‐1	IC_50_ = 120 mM	^164^, ^165^, ^166^, ^167^
	Zebularine	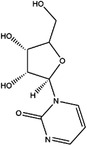	Preclinical	Breast, colorectal, liver and pancreatic cancer	MDA‐MB‐231, MCF‐7, SW620, SK‐Hep 1, PaCa‐44	IC_50_ = 10−100 μM	^168^, ^169^, ^170^
MTHFR	SAM	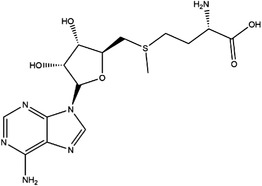	NA	NA	NA	Ki = 2.7 μM	^171^
MTHFS	5‐f‐THHF	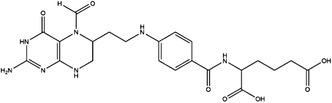	Preclinical	Breast cancer	MCF‐7	Ki = 0.7 μM	^172^

Abbreviations: ALL, acute lymphocytic leukaemia; CSCC, cutaneous squamous cell carcinoma; EMA, European Medicines Agency; NSCLC, non‐small cell lung cancer; US FDA, United States Food and Drug Administration.

### 4.4 ACHY inhibitors

Many inhibitors have been identified for ACHY, and most of them are adenine analogues. Li et al.^137^, ^173^, ^174^ developed a novel reversible inhibitor, methyl (S)‐4‐(6‐amino‐9H‐purin‐9‐yl)‐2‐hydroxybutanoate (DZ2002), for ACHY, which showed immune regulation abilities, including suppressing immune and inflammatory responses, blocking T‐cell activation and decreasing the generation of proinflammatory cytokines. Another ACHY inhibitor, 3‐deazaadenosine, also exhibited immunosuppressive and anti‐inflammatory activities by suppressing cytokine expression and monocyte adhesion.^138^, ^139^, ^175^ 3‐Deazaneplanocin A, derived from the antibiotic neplanocin A, is an immunomodulator in several tumours.^176^, ^177^, ^178^, ^179^ The irreversible ACHY inhibitors adenosine dialdehyde and adenosine deaminase perform immunosuppressive functions by suppressing the CD4^+^ T cell response.^148^, ^149^, ^150^, ^151^, ^174^, ^180^ d‐Eritadenine, isolated from the Lentinus edodes mushroom, and its analogues, including (S)‐9‐(2,3‐dihydroxypropyl)‐adenine, could significantly reduce the activity of ACHY.^140^, ^181^ Cu^2+^ inhibited the enzymatic activity of ACHY by inducing the release of NAD^+^ cofactors.^182^, ^183^ In addition, the antiviral drug ribavirin catalyses ACHY conversion to adenosine and Hcy and then blocks the activity of ACHY.^141^, ^142^, ^143^, ^144^, ^145^, ^146^ 3′‐keto aristeromycin and 2‐fluoroadenosine displayed the in vivo suppression activity of ACHY.^147^ Hao et al. screened thirteen compounds inhibiting ACHY by ChemMapper and SciFinder Scholar, which greatly increased the candidates for clinical research, and found that Compound 13 exerted considerable inhibitory effects on breast cancer and prostate cancer^152^, ^153^, ^154^ (Figure 3 and Table 4).

### 4.5 DNMT1 inhibitors

DNMT1 inhibitors contain the following types: nucleoside analogues and nonnucleoside analogues.^69^ Nucleoside analogues inhibited DNMT1 by imitating cytosine for DNA incorporation and degradation of DNMT1.^155^ United States Food and Drug Administration (US FDA)‐approved inhibitors include azacytidine, decitabine and guadecitabine, which have been used in the clinic.^69^, ^156^, ^157^ It was already confirmed that these drugs are associated with cancer immunotherapy by regulating the expression of PD1/PD‐L1, affecting the TME and activating cytotoxic T cells.^72^, ^184^ Furthermore, several novel compounds derived from natural products have been used in preclinical experiments, including antroquinonol D, isofistularin‐3, kazinol Q and harmine.^161^, ^164^, ^165^, ^166^, ^167^ These compounds showed different mechanisms in DNMT1 inhibition. Antroquinonol D competed with SAM for the same catalytic domain of DNMT1, resulting in DNMT1 suppression.^185^ Isofistularin‐3 and kazinol Q inhibited DNMT1 by binding to the DNA‐binding site and competing with cytosine binding, respectively.^161^, ^162^, ^163^ In addition, the cytidine analogue zebularine inhibited DNMT1 enzymatic activity.^168^, ^169^, ^170^, ^186^

Moreover, these compounds presented strong anticancer effects. Zebularine could inhibit the growth of various cancer cells. Moreover, zebularine promoted the infiltration of CD8^+^ T cells and natural killer cells into tumours enhanced the effects of cancer immunotherapy.^187^ Zebularine improved the cytotoxicity of CTLs and regulated the TME, which increased the response of cancer cells to immune checkpoint blockade.^188^, ^189^ Antroquinonol D inhibited the proliferation of various cancer cells, including breast cancer, lung cancer, glioma, pancreatic cancer colorectal cancer and liver cancer.^158^, ^159^, ^160^, ^190^, ^191^, ^192^ Isofistularin‐3 blocked the cell cycle phase, induced autophagy and then suppressed the growth of lymphoma cells.^162^ In addition, kazinol Q suppressed the proliferation of breast and prostate cancer cells by promoting apoptosis^163^ (Figure 3 and Table 4).

### 4.6 Other inhibitors

Bezerra et al.^171^, ^193^ discovered that SAM was an allosteric inhibitor of MTHFR, as it binds to the catalytic domain of MTHFR. Methenyltetrahydrofolate synthetase (MTHFS) catalyses 5‐formyltetrahydrofolate (5‐f‐THF) conversion to 5,10‐methenyltetrahydrofolate (5,10‐CH‐THF) and then participates in the synthesis of purine, which is upregulated in various cancers.^194^ Field et al.^172^, ^195^ found that 5‐formyltetrahydrohomofolate (5‐f‐THHF) was an effective inhibitor of MTHFS (Figure 3 and Table 4).

## 5 PROSPECTIVE AND CHALLENGES OF TARGETING 1C METABOLISM

1C metabolism could ensure that essential substances are available for the proliferation of cancer cells, such as purine, pyrimidine and amino acids.^3^ Moreover, 1C metabolism regulates epigenetic modification through the products of SAM and polyamines, which are generated from the methionine cycle and transsulphuration pathway.^196^ By influencing the synthesis of GSH and NADPH, 1C metabolism is crucial for controlling the redox steady state.^63^ Because of its participation in these biological functions, 1C metabolism regulates various downstream pathways that contribute to the progression of cancer. Thus, thoroughly understanding the function of 1C metabolism contributes to providing precise targets and specific pathways that are essential for cancer therapy. The successful application of anticancer drugs, such as pemetrexed and 5‐FU, provided strong evidence for the significance of 1C metabolism in oncotherapy. Excitingly, the finding of the interaction between 1C metabolism and T‐cell activation provided insight to improve immunotherapy.^197^ These studies demonstrated the importance of 1C metabolism enzymes in the development of cancer, providing promise for targeting 1C metabolism in the clinic in the future.

1C metabolism provides the precursors for nucleotide, protein, sphingolipid and phospholipid synthesis.^198^ The proliferation and growth of cancer cells rely, to some extent, on the availability of 1C units, suggesting that restricting the supplementation of 1C can yield more clinical benefits. Based on this theory, several 1C metabolism‐targeting drugs have been developed, including pemetrexed, methotrexate and 5‐FU. Recent studies have shown that these drugs are of great significance in cancer immunotherapy. For example, pemetrexed can present the inherent function of T cells and promote their activation in cancer, both in vitro and in vivo.^199^ In addition, pemetrexed enhances the anticancer effects of the PD‐1 inhibitor pembrolizumab.^200^ Thus, the development of 1C metabolism‐targeting inhibitors may be an effective approach to improve cancer immunotherapy.

1C units can be derived from serine, glycine, histidine, tryptophan and methionine, of which serine is the main source in cancer cells.^201^ Cancer cells increase their 1C supply by rapidly consuming exogenous serine. When exogenous serine is deficient, the SSP is activated. These two pathways promote the regulation of 1C metabolism in cancer. Thus, disruption of serine supplementation and synthesis may be useful for enhancing cancer immunotherapy.^202^ Reports have shown that serum serine levels can decrease by 50% in mice fed a serine‐deficient diet.^203^ Moreover, in xenograft models, this diet contributed to decreased tumour growth.^203^ Therefore, decreasing the level of 1C units by intervening in the diet may be appropriate for reducing the raw materials required for the pathway. Disrupting de novo serine synthesis is the second method that can be used for cancer therapy. In tumours with upregulated SSP activity, the depletion of SSP enzymes can suppress the proliferation and growth of cancer cells.^204^ Studies have revealed that inhibitors targeting PHGDH suppress the proliferation and reduce the growth of xenografts, especially in PHGDH‐dependent cancer cell lines.^205^ Therefore, targeting the SSP is a promising strategy for cancer treatment. Furthermore, cancers with amplified SSP enzymes are not readily influenced by a lack of exogenous serine; however, p53 depletion may aggravate this dependency.^206^ Thus, selecting appropriate methods according to the cancer type and other conditions is essential for cancer immunotherapy.

However, there are some unsolved questions that must be addressed. First, 1C metabolism is a very large complex network that not only supports the growth of cancer cells but also plays roles in normal cells. The traditional anticancer drugs 5‐FU and methotrexate are known as 1C metabolism targeting agents. However, severe side effects have limited their clinical usage due to undifferentiated attack on cancer cells and normal cells.^207^ Moreover, the emergence of drug resistance is also a common phenomenon in the clinic. Thus, enhancing the selectivity and sensitivity of targeting individual 1C metabolism enzymes is a future direction for drug development. 1C metabolism involves many biological reactions and products. A complete understanding of how the reactions and products might contribute to cancer is essential for targeting 1C metabolism. In addition, there is a lack of knowledge on the specific pathways of 1C metabolism, which are altered in cancer and determine the screening of therapeutic targets.

At present, research on the roles of 1C metabolism in the development of cancer has mainly focused on targeting specific enzymes. However, it is possible that cancer cells could rewire their metabolism to compensate when the function of 1C metabolism enzymes is disturbed. SHMT was applied to catalyse the conversion of serine and THF to glycine and 5,10‐methylenetetrahydrofolate. This reversible conversion contributed to nucleotide synthesis and DNA methylation regulation by providing 1C units. SHMT contains two isoforms, cytoplasmic SHMT1 and mitochondrial SHMT2. Although they belong to different compartments, the functions of SHMT1 and SHMT2 are the same.^53^ Thus, inhibiting the activity of SHMT1 or SHMT2 alone is not guaranteed to block cell metabolism, and cancer cells might be compensated by using the other. In addition, MTHFD enzymes were mainly used to catalyse the conversion of 5,10‐methylenetetrahydrofolate to 10‐f‐THF and 5‐methyltetrahydrofolate, which is essential to nucleotide synthesis and the methionine cycle. MTHFD enzymes have several forms, including mitochondrial MTHFD1L, MTHFD2 and MTHFD2L and cytoplasmic MTHFD1. Any enzyme changes could destroy the integrality of 1C metabolism. However, the extent of interference might not influence the progression of cancer because of the compensation mechanism. This phenomenon causes challenges when designing inhibitors. Given the urgency of this research, some methods might be used for solving the problems. On the one hand, it expands the range of targets that inhibitors act on under the premise of the accuracy of inhibitors. For example, SHIN1 is a dual inhibitor of SHMT1 and SHMT2, which could influence the related functions in both the cytoplasm and mitochondria simultaneously. In addition, studies have revealed that THF conjugates cannot pass through the mitochondrial membrane.^208^ Instead, 1C units existing in mitochondria were ultimately converted to formate, which could be transported into the cytoplasm.^4^ For this reason, the cytoplasmic enzymes MTHFD1 and SHMT1 might be more valuable targets. Therefore, these problems should be addressed before drugs targeting 1C metabolism can be exploited.

Given the complexity of immune cell metabolism and immune response regulation, defining the role of 1C metabolism in immunotherapy is challenging. Moreover, the interaction between 1C metabolism and various cellular reactions cause further difficulties. 1C metabolism secreted metabolites into the TME and rebuilt the TME. Serine participated in T‐cell activation, bioenergetics and effector function.^209^ Folic acid is essential for the survival of Treg cells and is accompanied by high expression of the folic acid receptor.^210^ These studies emphasised the importance of understanding how 1C metabolism affects immune homeostasis. Furthermore, the regulation of 1C metabolism was cell‐type and context dependent.^211^ Thus, a detailed understanding of the mechanistic connection between 1C metabolism and immune cells would offer new insights for cancer immunotherapy. In addition, the role of different compartments, including cytoplasm and mitochondria, in which 1C metabolism activation influences immune cell function should be illustrated. In summary, searching for specific metabolic checkpoints to improve the availability of cancer immunotherapy is still difficult.

## 6 CONCLUSION

In the last decade, the exploration of the roles of metabolic pathways in cancer biology has attracted the attention of researchers. In particularly, 1C metabolism contributes to the proliferation and growth of cancer cells by providing a precursor material for nucleotides and amino acids. In addition, there is exist an association between 1C metabolism and epigenetics as well as the mitochondrial redox state. Thus, a comprehensive understanding of 1C metabolism and its metabolic enzymes could enable more precise targeting of specific pathways essential for the occurrence and development of cancer. However, given the importance of 1C metabolism in healthy cells, including immune cells, serious adverse effects are a common problem that hinders the exploitation of inhibitors targeting 1C metabolism, which needs to be overcome in the future.

Recently, immunotherapy has become a powerful clinical strategy for cancer treatment. However, the occurrence of immune escape and low response rates in patients prevent immunotherapy from achieving maximum effectiveness. Discovery of the relationship between 1C metabolism and immunotherapy may contribute to solving these problems. SHMT2 and MTHFD2, regarded as oncogenes, are significantly upregulated in various cancer types and promote cancer cell survival. Thus, SHMT2 and MTHFD2 are potential targets for cancer treatment due to their specific expression and prognostic value, which attract considerable attention. Moreover, SHMT2 and MTHFD2 may influence the curative effect of immunotherapy by regulating the expression of several immune checkpoints and the TME. However, several questions remain unanswered. For example, the expression of MTHFD2 is positively associated with T cell activation, and it is unclear whether MTHFD2 deletion benefits immunotherapy. Therefore, a deeper understanding of the main functions and mechanisms of 1C metabolic enzymes in immune cells will provide a new strategy for improving immunotherapy.

The relationship between 1C metabolism and immunotherapy is yet to be fully understood, and the mechanisms by which 1C metabolic enzymes regulate immunotherapy remain unknown. Whether any of the new functions of these enzymes in immunotherapy will lead to further clinical benefits requires further investigation. The application of new technologies, such as integrative bioinformatics, metabolomics and computational models, will help us uncover many other unknown mechanisms that connect 1C metabolism and immunotherapy in cancer. In addition, a further understanding of 1C metabolic pathways will contribute to the development of new drugs. Therefore, achieving these goals has the potential to improve cancer treatment and allow us to achieve the greatest benefit in specific patient populations.

## AUTHOR CONTRIBUTIONS

M. H. G., P. H., J. J. X., X. X. R. and X. W. collected the related papers. X. X. R. and X. W. drafted and wrote the manuscript. G. W. Z., S. S. W., Q. Y. W., M. N. Y. and T. X. revised the manuscript. All authors have read and approved the final manuscript.

## CONFLICT OF INTEREST STATEMENT

The authors declare that they have no competing interests.

## ETHICS SATEMENT

Not applicable.

## Supporting information




Supporting information

Supporting information
