# Supplementary figures and images for: Targeting one‐carbon metabolism for cancer immunotherapy
Source: Clin Transl Med. 2024 Jan 27;14(1):e1521. doi: 10.1002/ctm2.1521 (PMC10819114; doi:10.1002/ctm2.1521)

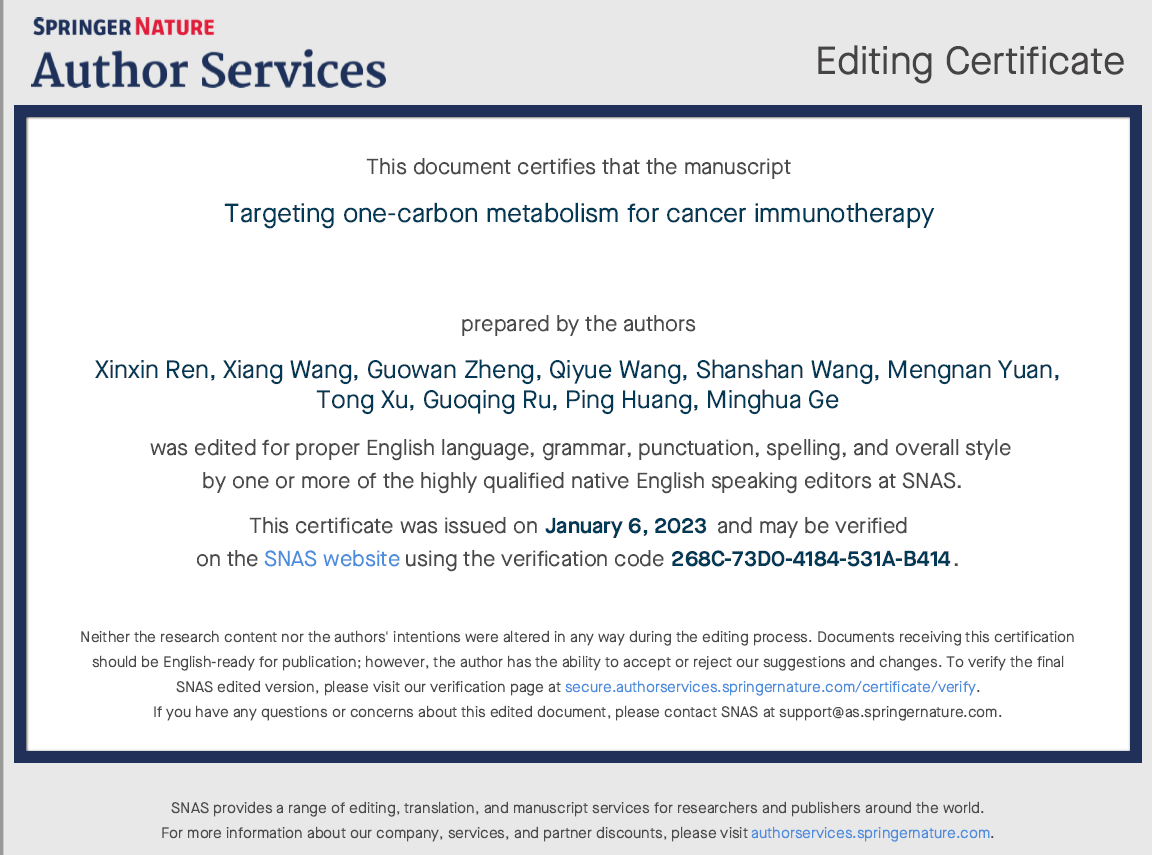

Supplement: Supplementary file 1 — Supporting information [file CTM2-14-e1521-s002.docx]

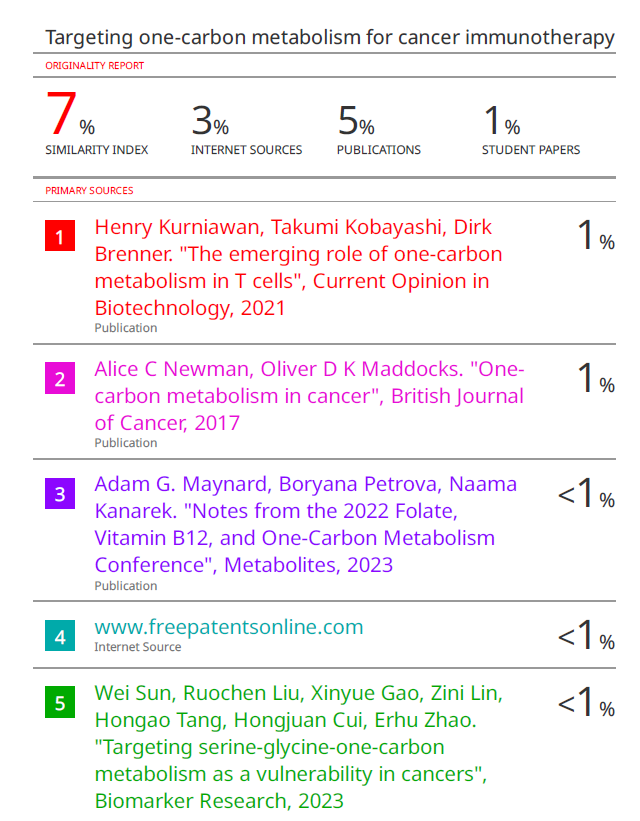

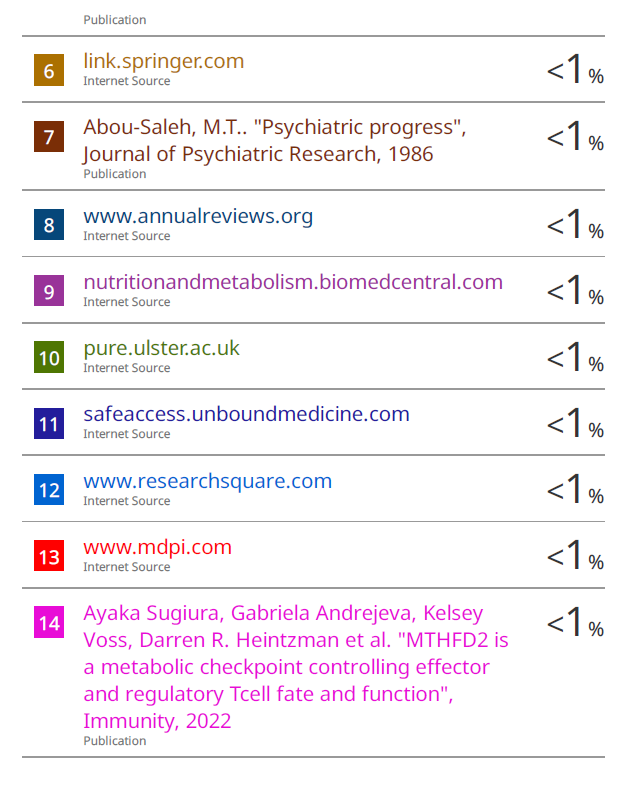

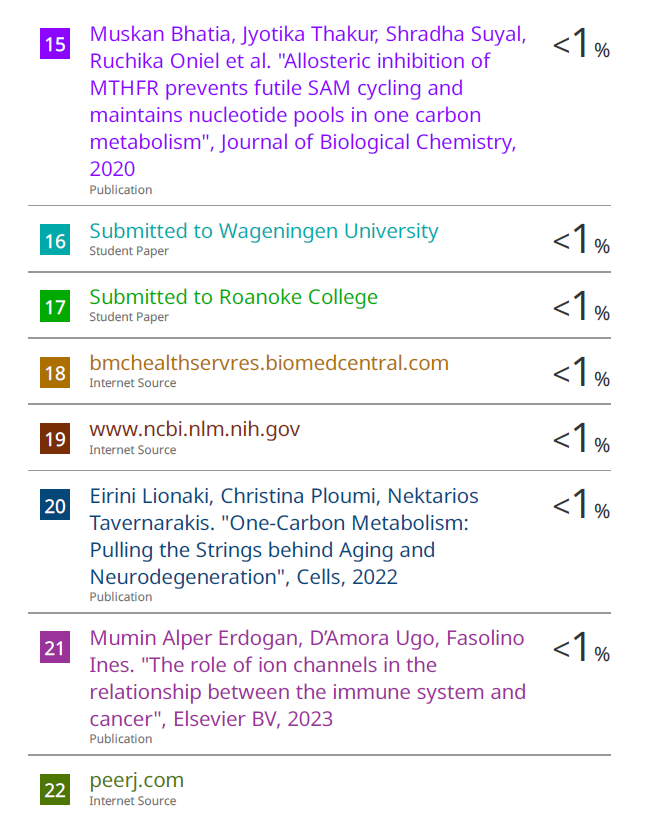

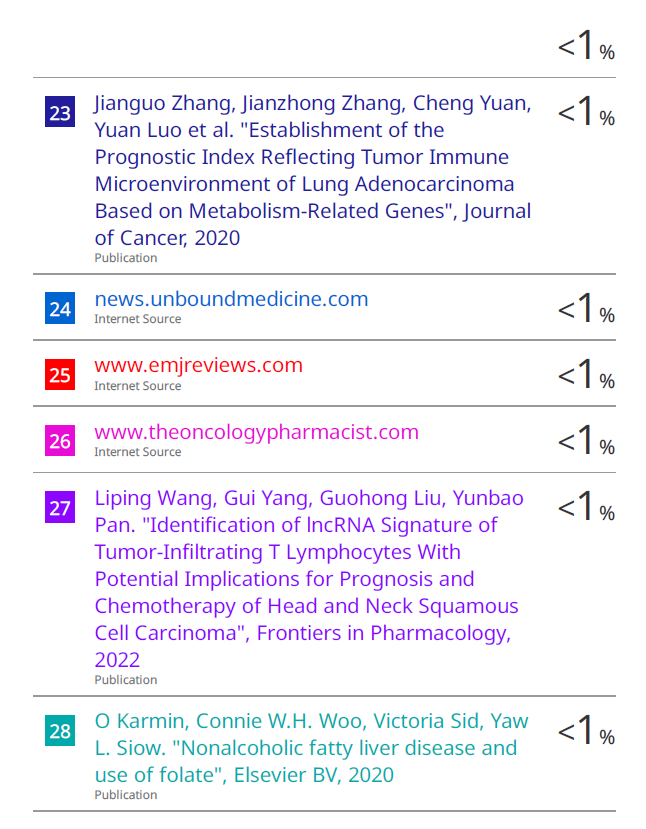

Supplement: Supplementary file 2 — Supporting information [file CTM2-14-e1521-s001.docx]
